# Experimental Investigation and Molecular Dynamics Simulation on the Anti-Adhesion Behavior of Alkanethiols on Nickel Insert in Micro Injection Molding

**DOI:** 10.3390/nano11071834

**Published:** 2021-07-14

**Authors:** Can Weng, Jiachen Chen, Jin Yang, Mingyong Zhou, Bingyan Jiang

**Affiliations:** College of Mechanical and Electrical Engineering, Central South University, Changsha 410083, China; canweng@csu.edu.cn (C.W.); csuchenjiachen@csu.edu.cn (J.C.); yj20141116@csu.edu.cn (J.Y.); jby@csu.edu.cn (B.J.)

**Keywords:** anti-adhesion, self-assembled monolayers, molecular dynamics simulation, micro injection molding

## Abstract

Due to the adhesion between the polymer melt and nickel (Ni) mold insert in the micro injection molding process, deformation defects frequently occur when the microstructures are demolded from the insert. In this study, self-assembled alkanethiols were applied to modify the surface of Ni mold insert to reduce its surface energy. Experimental trials were undertaken to explore the effect of alkanethiols coating on the replication quality. After that, molecular dynamics (MD) simulation was then used to investigate the adhesion behavior between the self-assembled coating and polypropylene (PP) by establishing three different types of alkanethiol material. The interaction energy, the potential energy change and radial distribution function were calculated to study the anti-adhesion mechanism. Experimental results show that all the three coatings can effectively decrease the adhesion and therefore promote the replication fidelity. It is demonstrated in MD simulation that the adhesion mainly comes from the van der Waals (vdW) force at the interface. The arrangement of sulfur atom on the Ni surface results in different absorbing behaviors. Compared with that of the PP–Ni interface, the interfacial energy and adhesion work after surface treatment is significantly reduced.

## 1. Introduction

Devices with micro/nano structures, which are widely used in adaptive optics [1], anti-counterfeiting coatings [2], bionic surface [3] and microfluidic chips [4] are playing an important role in the development of modern science and technology. The quality of micro/nano structures is the decisive factor for characterizing its function. Injection molding technology is a commonly used method to fabricate plastic products with complex shape and size [5], due to the benefit of high precision [6], high efficiency [7], high speed [8], low cost [9], and potential in mass production [10].

The injection molding process mainly includes the following steps: injecting, filling, packing, cooling and demolding. Plastic products with high replication quality can be obtained by carefully controlling the conditions in each step. When the structure is down to micrometer or nanometer scale, the replication quality will directly affect the function and characteristics of products [11,12]. Therefore, researchers have carried out much experimental research on the quality of micro/nano-structures, especially during the filling or demolding process. Zhang et al. [13] studied the effect of mold surface roughness on the cavity filling of polymer melt in micro injection molding. It was found that the mold surface roughness had a resistance effect on the cavity filling of polymer melt. During the demolding process, the polymer product is mainly affected by the friction force, adhesion and thermal shrinkage, thus leading to possible defects such as bending, necking and even fracture in micro/nano-structures. Sasaki et al. [14] studied the relationship between the demolding force of different materials and the surface roughness of the mold insert during the demolding process. When the surface roughness was below 0.1 μm, the main factor affecting the demolding force was the vdW force, which came from the interfacial interaction between the metal insert and polymer material.

During the demolding process, the polymer melt is easy to adhere to the mold insert when a high packing pressure is applied in the cavity. A mold insert with an excellent anti-adhesion property can reduce the cleaning process and further increase the service life for the whole mold. Lee et al. [15] deposited n-dodecyl mercaptan as an anti-adhesion layer on the Ni template in the nano-replication process for optoelectronic applications. Yoon et al. [16] studied the effect of deposition of FOTS coating on the surface of Si mold insert on the microstructure array parts in an injection molding process. The results showed that the surface coating improved the molding quality of microstructures in replication fidelity, aspect ratio and surface roughness. Kwon et al. [17] studied the effect of thickness of self-assembled FOTS coating on the imprinting quality by a molecular dynamics method. The self-assembled alkanethiols is also an alternative in anti-adhesion application, since the alkanethiol monolayers can be effectively adsorbed on the metal surface. Tsvetanova et al. [18] studied the work function of dodecyl mercaptan self-assembly on a silver surface and compared the induced work function variations. Chen et al. [19] studied the effects of surface composition of mixed self-assembled coating on the wettability of a silver matrix by using mixtures of fluoroalkyl mercaptans and carboxyl terminated mercaptans with different chain lengths.

Although many studies on surface modification have been undertaken, the modification mechanism in micro/nano-scale, especially the interfacial interaction between the substrate and the coating layer, still needs much more investigation. Recently, molecular dynamics (MD) theory has been used in the analysis of the polymer molding process and the interaction mechanism of the polymer–metal interface at the atomic level. J. Pina et al. [20] overcame the scale limitation of computational fluid dynamics and exposed a molecular dynamics method to predict the replication of nanocavities. Zhou et al. [21] analyzed the snapshots, densities and gyration radiuses in the filling process and influences of packing parameters on geometrical morphology by constructing a molecular dynamics simulation and experimental trial for injection molding nanopillars on the polymethylmethacrylate surface. Kang et al. [22] established a self-assembled coating of trichloroctadecylsilane (ODTS) and perfluorooctyl trichlorosilane (FOTS) on the SiO_2_ surface. It was found that the surface energy and adhesion energy in the nano imprinting process decreased significantly when the mold insert surface had an anti-adhesion coating. The FOTS coating with fluorine element had a better anti-adhesion performance than ODTS coating.

In this paper, a self-assembled coating with low surface energy was deposited on the mold surface. With the help of anti-adhesion treatment, the replication quality of injection-molded microstructures was improved. According to the experimental results, the atomic models of self-assembled molecules on the insert surface were built. The adsorption behaviors of different interfaces were investigated by MD method [23,24,25]. Moreover, the anti-adhesion mechanisms of the polymer-alkanethiols interface in different arrangements were explored by analyzing the morphological evolution, potential energy, interaction energy, adhesion work and the radial distribution function at the interface comprehensively.

## 2. Experimental Trials

### 2.1. Anti-Adhesion Coating Preparation

To reduce the adhesion of polymer-mold interface and the demolding defect of injection-molded microstructures, a self-assembled coating with low surface energy was deposited on the mold surface. For the mold insert, Ni metal was selected because insert with micro/nano-structures were usually fabricated by Ni electroforming process. In this study, decanethiol (DT), decanedithiol (DDT) and perfluorodecanethiol (PFDT) were prepared as the chemical solution to form anti-adhesion layers. When the Ni shim was immersed in the chemical solution at 82 °C, the sulfur atom at the head of alkyl mercaptan molecule could form a chemical bond with the Ni atom.

### 2.2. Micro-Injection Molding Process

The Ni insert with anti-adhesion coating was then installed in the fixed mold. It is known that polypropylene (PP) is one of the commonly used materials in injection molding process. In this study, PP (HD120MO, Borealis AG, Vienna, Austria) was selected as the experimental material. The precision injection molding machine (Sodick TR05EH2, Kanagawa, Japan) was used to fabricate the polymer products with micro-pillar arrays. The main process parameters of the injection molding process are shown in Table 1. The surface morphology and the demolding quality of microstructures were explored by an atomic force microscope (Veeco NanoManVS/Multimode, Santa Barbara, American) and the Laser Scanning Microscope (Carl Zeiss LSM 700, Oberkochen, Germany), respectively.

### 2.3. Effect of Anti-Adhesion Coating on the Replication Quality

**Figure 1:**
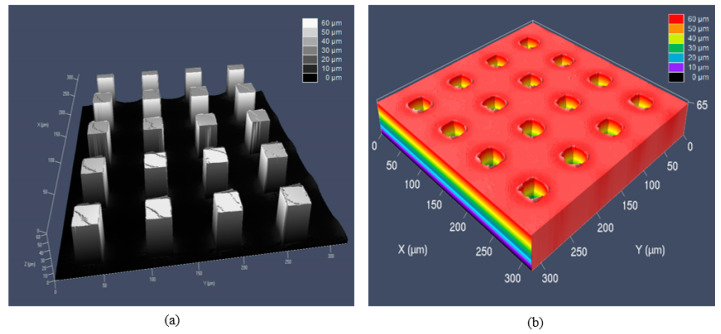
Microstructures on the (**a**) silicon master and (**b**) electroformed Ni shim.

**Figure 2:**
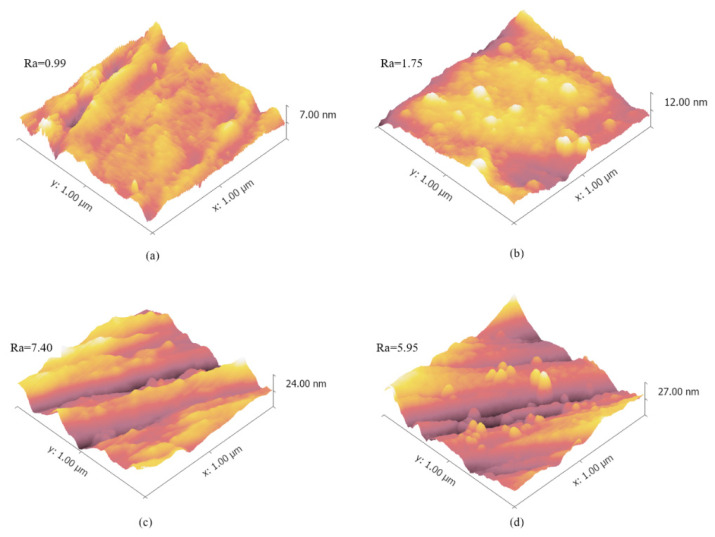
Atomic force microscopy (AFM) images of the surface morphology of mold insert before and after anti-adhesion treatment: (**a**) no coating, (**b**) decanethiol (DT), (**c**) decanedithiol (DDT) and (**d**) perfluorodecanethiol (PFDT).

The silicon master and the electroformed Ni shim as the mold insert were shown in Figure 1. The side length of micro-pillar was 23.2 μm and the height was 60.6 μm, with a distance between two pillars of 60.0 μm, which were measured by LSM. It was shown that the microstructures on the silicon master were well transferred to Ni mold insert. Figure 2 is the AFM images for the surface morphology of mold insert after the anti-adhesion treatment. It can be seen that the surface was relatively smooth, with low surface roughness. There was no obvious damage to the micro-pillars and the whole surface of the mold insert.

**Figure 3:**
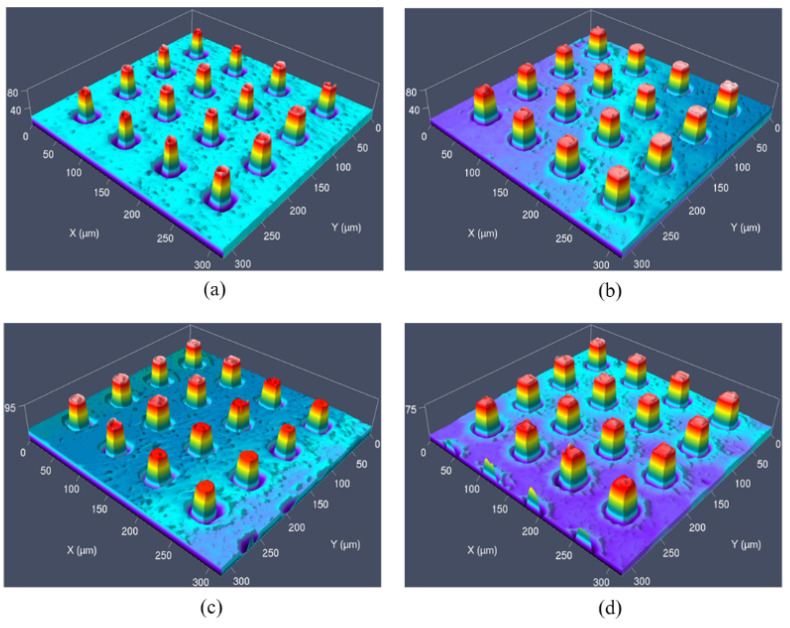
The injection-molded micro-pillars without and with anti-adhesion coating treatment: (**a**) no coating, (**b**) DT, (**c**) DDT, (**d**) PFDT.

Figure 3 shows the injection-molded micro-pillars without and with anti-adhesion treatment. When the mold insert was without any surface treatment, the replication quality of micro-pillar was much worse than that with anti-adhesion coating by DT, DDT and PFDT. The sunken defects on the top surface of micro-pillars almost disappeared and the shape became plump thanks to the anti-adhesion coating. It was demonstrated that the introduction of DT, DDT and PFDT coating could effectively decrease the adhesion and promote the replication fidelity. Therefore, in the following study, the interfacial interaction model of PP-alkanethiols will be constructed and an anti-adhesion mechanism at the interface would be comprehensively analyzed at the atomic scale by using MD simulation.

## 3. Simulation Models and Methods

### 3.1. Materials and Model Construction

In this simulation work, the same interface model as the actual condition was constructed.

**Figure 4:**
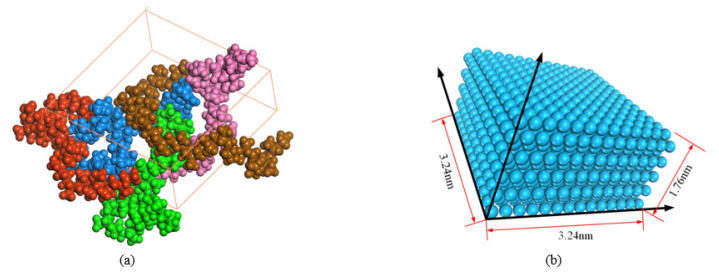
Atomic models for (**a**) PP and (**b**) Ni layer.

By constructing PP with different monomer numbers and calculating their cohesive energy density, the single chain with a polymerization degree of 30 was selected. After annealing treatment, the amorphous PP system with periodic boundary conditions was constructed. The main parameters for the PP layer are shown in Table 2. In order to observe the movement of molecular chains at the three interfaces, different colors were selected to distinguish the five chains. The steepest descent method [26], the conjugate gradient method [27], and the Newton method [28] were used, respectively, to minimize the energy of PP polymer, as shown in Figure 4a. For mold insert, the Ni layer with (1 0 0) surface was established, as exhibited in Figure 4b. It should be noted that the dimensions in length and width should be the same for both layers, so that the interface model could be established.

**Figure 5:**
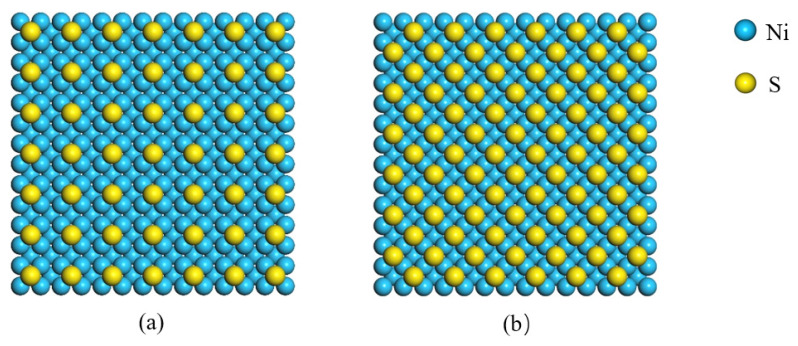
Arrangements of sulfur atoms adsorbed on Ni surface: (**a**) P (2 × 2) and (**b**) C (2 × 2).

When sulfur atoms are adsorbed on the surface of Ni, adsorption modes are different. [29]. For anti-adhesion layer, the adsorption of sulfur atom to form P (2 × 2) and C (2 × 2) phase on Ni with (1 0 0) surface was selected, as shown in Figure 5. The Ni atom in the top surface was correspondingly replaced by sulfur atom and the location of sulfur atom was moved according to the adsorption of the sulfur atom on Ni with (1 0 0) surface. The height of sulfur atom from Ni surface was 0.131 nm, and the length of the S–Ni bond was 0.22 nm. Three kinds of self-assembled molecule were obtained by grafting sulfur atoms. After the energy minimization process, the interface model was established by assembling the anti-adhesion layer and the PP layer. In order to avoid the influence of a periodic boundary in the height direction, a vacuum layer with a thickness of 3.0 nm was set above the PP layer.

In this paper, the coverage (*α*) was defined as the ratio of the number of sulfur atoms adsorbed on the *Ni* surface (*Ns*) to the number of *Ni* atoms on the *Ni* surface (*N_Ni_*), as shown in Equation (1):

(1) $$\alpha =\frac{N_{S}}{N_{Ni}}\times 100\%$$

where sulfur atoms were adsorbed on *Ni* surface in P (2 × 2) and C (2 × 2) arrangement, the coverages were 25% and 43%, respectively.

### 3.2. Force Field and Potential Function

Compass force field was used in this work to describe the force field in organic and inorganic molecular systems [30,31]. It is composed of bond stretching potential, angle bending potential, dihedral angle torsion potential, and non-bonded potential [32,33,34]. The classical Lennard–Jones 12-6 potential and Coulomb potential were adopted to describe the vdW interaction and the electrostatic interaction energy between non-bonded atoms. The non-bonded potential function is shown in Equation (2), as follows [35]:

(2) $$U_{nonbonded}=4\varepsilon \left[{\left(\frac{\sigma }{r}\right)}^{12}-{\left(\frac{\sigma }{r}\right)}^{6}\right]+\frac{q_{i}q_{j}}{r}(r<r_{c})$$

where ε, σ, r, q, and $r_{c}$ are the constants of non-bonded interaction, equilibrium distance, real distance between two atoms, the charges of atoms, and the cutoff distance. The cutoff distance of Lennard–Jones potential and Coulomb potential was set to 1.60 nm.

### 3.3. Simulation Process

To keep the number, the volume, and the temperature of the whole system constant, a dynamics simulation progress was undertaken in the NVT ensemble, with a total simulation time of 500 ps and a time step of 1.0 fs. Moreover, the periodic boundary condition was set during the simulation. The temperature was set to consistent with the actual condition in alkanethiols coating process, which was controlled by Nose thermostat [36]. The trajectories of atoms were recorded every 1000 steps. During the whole simulation process, the Ni layer was regarded as a rigid body by fixing the location of the whole atoms. Since the Compass Force Field did not contain the bond parameters for the S–Ni bond, the sulfur atoms were also fixed to ensure their initial position. All the works were done with Materials Studio (MS), which could be used to build atomic models and run MD simulations.

## 4. Results and Discussion

### 4.1. Evolution of Molecular Chains at the Interface

**Figure 6:**
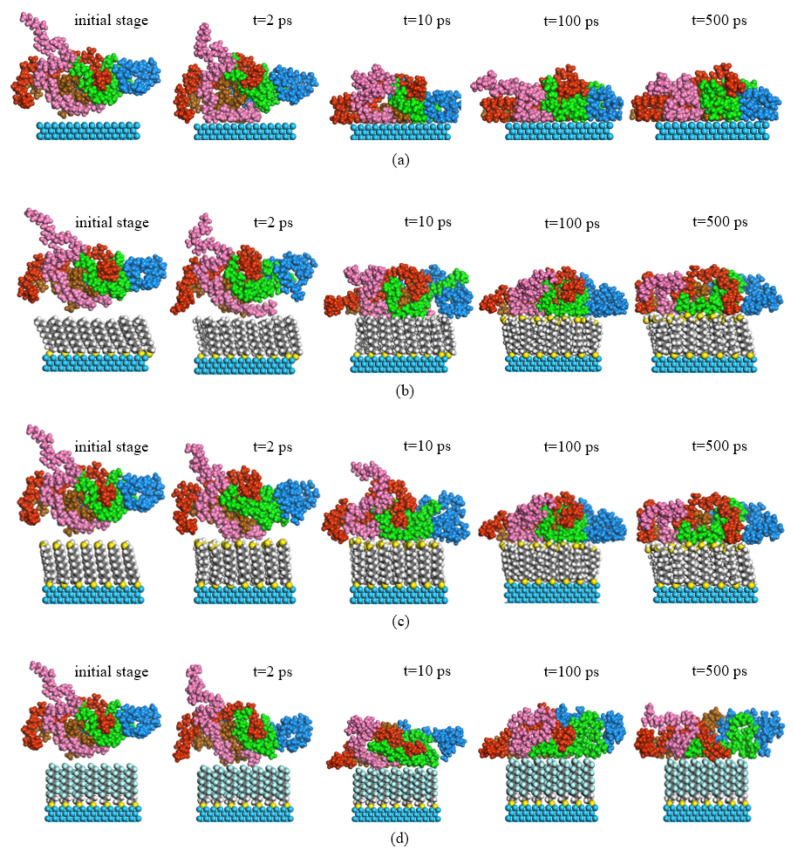
Morphological evolution of PP without coating and PP with coating in P (2 × 2) arrangement: (**a**) no coating, (**b**) DT, (**c**) DDT and (**d**) PFDT.

During the dynamic optimization process, the morphology evolution diagrams of PP chains in P (2 × 2) arrangement at three interfaces and the PP–Ni interface were analyzed, as shown in Figure 6. At the initial moment, the PP chains were in a disordered state, far away from the surface of the self-assembled coatings. After 2 ps, the chains removed the entanglement and began to move downward, with some atoms in the PP layer close to the membrane. The molecular chains in the PP–DDT and PP–PFDT interfaces were close to the self-assembled surface, while such a phenomenon was not observed in PP–DT interface. It could be inferred that there was an interaction force between PP and alkanethiols molecules. Most of the PP molecules in all three interfaces were adsorbed on the surface of monolayers at 10 ps. After that, the morphology of the PP chain changed slightly.

In the initial stage of dynamic optimization simulation, coatings of alkanethiols were arranged on the Ni surface, forming a certain angle to the surface. This was due to the strong vdW among alkanethiols, at which strength alkanethiols stand on the Ni surface and form dense self-assembled coatings. The order of angles between alkanethiols and Ni surface were as follows: PFDT, followed by DDT and DT. After the simulation of dynamic optimization, the angles between them were close to 90°. It was caused by the adhesion that disrupts the arrangement of alkanethiols on the Ni surface. It is shown in Figure 6c that the arrangement of PFDT molecules was not affected by the interfacial adhesion force and the angle to the Ni surface remained unchanged during the whole process.

**Figure 7:**
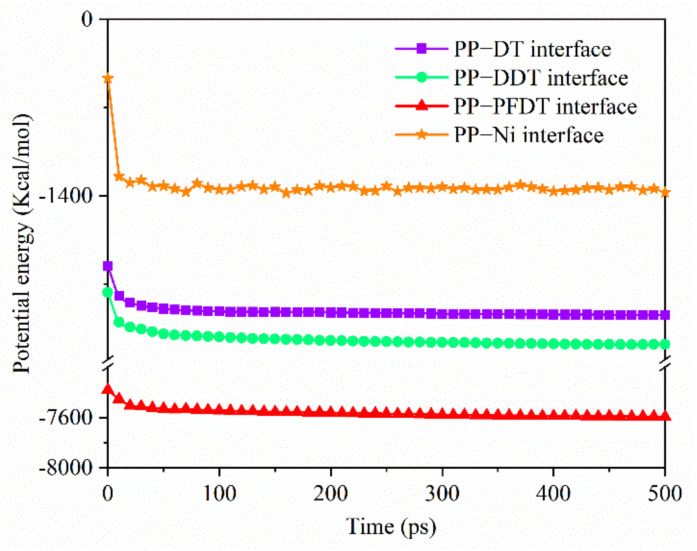
Potential energy evolution of PP–Ni surface and PP–DT/DDT/PFDT surfaces in P (2 × 2) arrangement.

The total potential energy changes of four interface models in dynamic optimization simulation are shown in Figure 7. It was seen that the total potential energy of the four interfaces decreased sharply in the first 10 ps, indicating that the configurations of PP chains were greatly changed to optimize the structure. After that, the total potential energies decreased smoothly. This meant that the configuration of PP chains had no obvious change.

### 4.2. Anti-Adhesion Mechanism in P (2 × 2) Arrangement

In order to analyze the adhesion force in three interface models, the formula of interfacial adhesion force was introduced, as shown in the Equation (3), as follows [37]:

(3) $$E_{work}=\frac{-E_{interaction}}{A_{c}}=\frac{-[E_{total}-\left(E_{PP}+E_{mold}\right)]}{A_{c}}$$

where $E_{interaction}$, $E_{total}$, $E_{PP}$, $E_{mold}$ and $A_{c}$ were the interfacial interaction energy between PP and mold material, the total energy of the interfacial model, the energy of PP, the energy of the mold material, and the surface area of the mold material, respectively. In this work, the interfacial energies come from the contribution of the intermolecular interaction since no chemical bond is formed at the interface. The interface energy refers to the energy between the PP and the mold insert layer. As shown in Table 3, the interaction energy was calculated at the simulation time of 500 ps and mainly came from the vdW energy. The values of interaction energy were negative, which meant there were adhesion interactions between PP molecule and alkanethiols membrane. By comparing the absolute value of the interaction energy, the PP–Ni interface was the largest, followed by PP-PFDT, PP-DDT and PP-DT interfaces. This meant that the surface coating of alkanethiols could significantly decrease the interaction energy and reduce the adhesion work of the insert, which would help to reduce the adhesion of polymer molecules to the insert surface during the demolding process.

The type of functional groups determined their electrical properties and further affect the electrostatic force between molecules. For the PP-DT interface, the electrostatic energy was positive, indicating that there was a repulsion force between PP and the decyl mercaptan molecule, which would prevent the adsorption of PP molecule on the Ni surface. Moreover, the side chain in the PP layer was mainly non-polar methyl groups and the end of the decyl mercaptan chain was also methyl groups with electron withdrawing character. When the PP molecule was gradually attracted by vdW force and moves towards the DT surface, electrostatic repulsion force was generated. Because the vdW force of the PP–DT interface was much higher than the electrostatic repulsion force, PP molecules overcame the electrostatic force at the interface and were gradually adsorbed on the surface. For the PP–DDT and PP–PFDT interfaces, the electrostatic energies were negative, which indicated that electrostatic attraction promoted the adsorption of PP molecules on the Ni surface. Also, the mercapto and trifluoromethyl groups at the end of the DT and PFDT layer were electron withdrawing. The trifluoromethyl groups had a more significant electronegativity than the mercapto group. Therefore, the electrostatic attraction of the PP–PFDT interface was more obvious, resulting in the maximum adhesion work.

**Figure 8:**
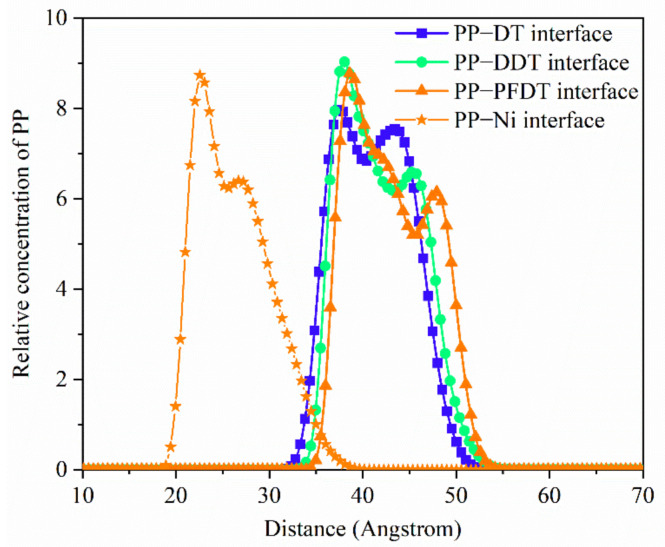
Relative concentration distribution of PP at four interfaces after 500 ps in the P (2 × 2) arrangement.

In order to explore the distribution of the PP molecules on alkanethiols surface, the relative concentration distribution in the PP layer was investigated, as shown in Figure 8. It was seen that the relative concentration reached the peak near the surface of the alkanethiols coating, and then was decreased slowly. The relative concentration at the PP–DDT interface was the highest and both concentrations at the PP–DDT interface and PP–PFDT interface were higher than that at the PP–DT interface. This was due to the repulsion force between PP and decyl mercaptan groups and the overall low adhesion energy at the PP–DT interface. The regulation in relative concentration distribution of PP molecules was basically consistent with interaction energy at the interface.

Radial distribution function (RDF) analysis of the interface model was carried out to explain the interaction mechanism. The RDF represents the probability density of the presence of another particle at the distance of r, which is expressed as the ratio of local density to total average density. The specific calculation, Equation (3), is as follows:

(4) $$g\left(r\right)=\frac{n\left(r\right)}{\rho_{0}V}\approx \frac{n\left(r\right)}{4\pi r^{2}\rho_{0}\delta_{r}}$$

where r, $\delta_{r}$, n(r) and $\rho_{0}$ are the distance from the radius of the central atom, the thickness, the number of particles in a spherical shell and the number density in ideal crystal. Before calculation, carbon atoms, hydrogen atoms and methyl groups in the PP molecule near the interface were labeled as C(PP), H(PP), and CH_3_(PP), respectively. The carbon atoms, hydrogen atoms and methyl groups at the terminal interface of DT molecules were also labeled as C(DT), H(DT) and CH_3_(DT). The sulfur atoms, hydrogen atoms and sulfhydryl groups at the end interface of DDT in the PP–DDT interface were labeled as S(DDT), H(DDT), and SH(DDT). The carbon atoms, fluorine atoms and trifluoromethyl groups at the terminal interface of PFDT in the PP–PFDT interface were labeled as C(PFDT), F(PFDT), CF_3_(PFDT).

**Figure 9:**
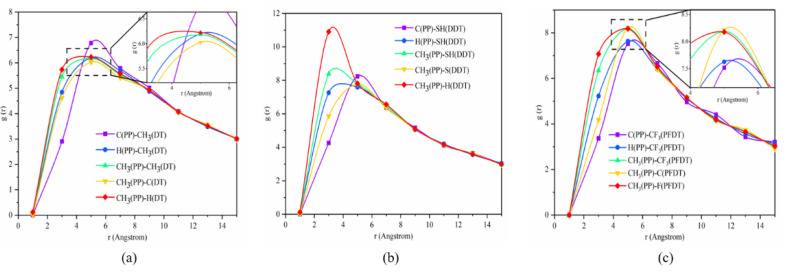
Radial distribution functions of the three interfaces in P (2 × 2) arrangement: (**a**) PP–DT, (**b**) PP–PFDT and (**c**) PP–PFDT.

The radial distribution functions of the three models are shown in Figure 9. Overall, the peak of the radial distribution function of the PP–DT interface was not obvious, which indicated that the intermolecular interaction at the interface was relatively weak. For the PP–DT interface, as shown in Figure 9, the peak value of RDF for CH_3_(PP)-CH_3_(DT) was 6.18, at 0.5 nm. From the radial distribution functions of C(PP)-CH_3_(DT), it can be seen that the interaction between C in the PP layer and methyl groups in the DT layer was the main reason. The g(r) peak value of CH_3_(PP)-H(DT) was larger than that of CH_3_(PP)-C(DT), which indicated that PP molecules mainly interact with hydrogen atoms at the terminal interface of DT.

The PP–DDT interface had a similar distribution to that of the PP–DT interface, as shown in Figure 9b. The interaction intensity of CH_3_(PP)-H(DDT) was significantly higher than that of CH_3_(PP)-S(DDT), with a peak value of 11.18. Since the methyl groups in the PP molecule were electron donating and the mercapto groups in DDT were electron withdrawing, intermolecular interactions between the methyl groups in the PP molecule and the hydrogen atom of the DDT molecule were generated, including the vdW attraction and electrostatic repulsion force.

It was shown in Figure 9c that the interaction intensity between methyl groups of the PP molecule in the PP–PFDT interface and fluorine atom in PFDT was greater than that of the carbon atom. The electrostatic attraction of the PP–PFDT interface might be formed by the interaction between the electron-donating methyl groups in the PP layer and the electron-withdrawing trifluoromethyl groups in PFDT. By comparing the radial distribution functions of the three interface models, it was observed that the g(r) peak values of the PP–DDT interface and the PP–PFDT interface were higher than that of the PP–DT interface, which indicated that the interaction energy of the PP–DT interface was the smallest and PP is relatively difficult to adsorb on the DT surface.

### 4.3. Anti-Adhesion Mechanism in C (2 × 2) Arrangement

**Figure 10:**
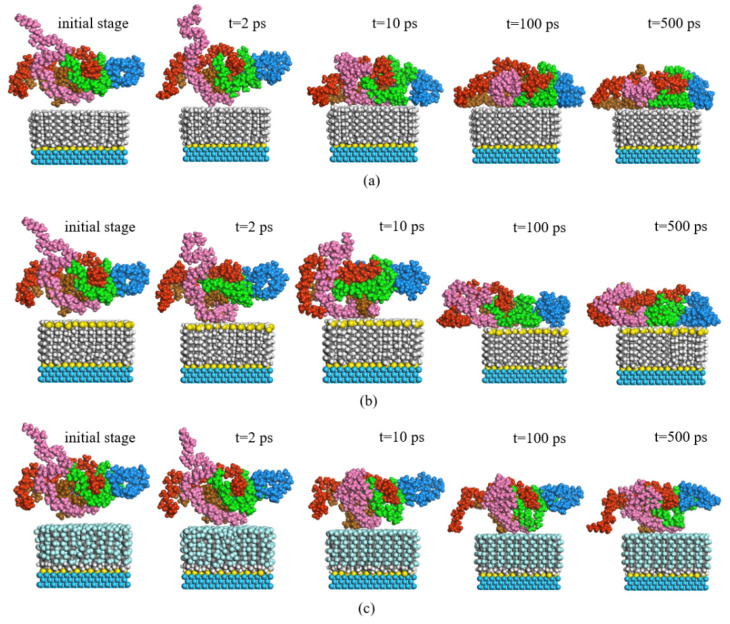
Morphological evolution of PP in C (2 × 2) arrangement: (**a**) DT, (**b**) DDT and (**c**) PFDT.

In the case of the sulfur atoms in C (2 × 2) arrangement, the morphology evolutions of PP chains during the dynamic optimization process are shown in Figure 10. In general, the configuration changes in the C (2 × 2) arrangement were like that in the P (2 × 2) arrangement. As shown in Figure 10, the difference was that only a few PP molecules were adsorbed on the self-assembled PFDT surface, while most molecules were far away from the surface. It was demonstrated that PP molecules were not easy to enrich on the PFDT surface in the C (2 × 2) arrangement. In addition, by observing the morphology of the three self-assembled molecules on the Ni surface, it was found that all the molecules were perpendicular to the Ni surface, forming a dense and highly-ordered alkanethiols coating.

**Figure 11:**
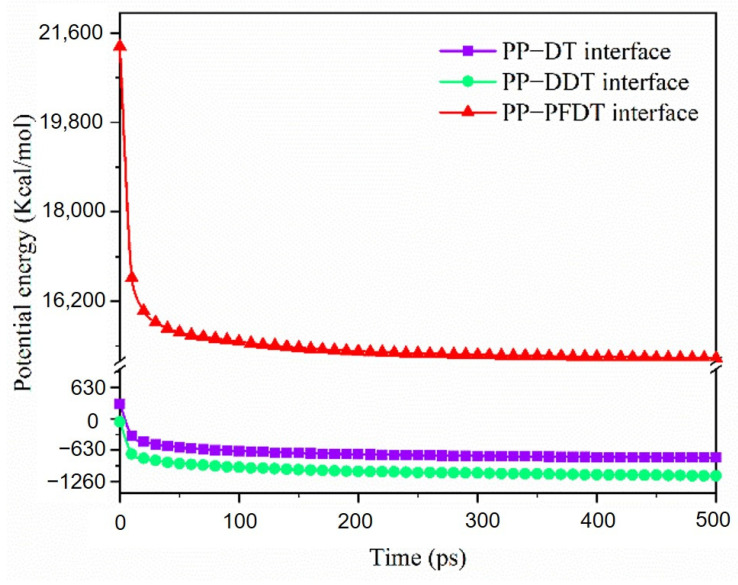
Potential energy evolution of three interface models in dynamic simulation in the C (2 × 2) arrangement.

The total potential energies of three interface models during the dynamic simulation process are shown in Figure 11. The total potential energy of the self-assembly model in the C (2 × 2) arrangement were higher than that in the P (2 × 2) arrangement indicating that the adsorption of PP molecules on the surface of C (2 × 2) self-assembly was not stable enough. However, the total potential energy of the PP–PFDT interface was still positive after 500 ps, significantly higher than that of the PP–PFDT interface with the P (2 × 2) self-assembly mode, which could be the reason why the adhesion work of the PP–PFDT interface decreases and PP was not easy to adsorb on the PFDT surface. Compared with the PP–PFDT interface, both the PP–DT and PP–DDT interfaces had a relatively stable interface adsorption model. Therefore, when alkanethiols were self-assembled on the Ni surface in the C (2 × 2) arrangement, the PFDT-modified Ni insert would have better demolding performance than DT and DDT.

The interaction energy and adhesion work in three kinds of interface model were calculated at the simulation time of 500 ps, as shown in Table 4. Compared with the PP–Ni interface, the interfacial interaction energy between the self-assembled alkanethiols monolayer and the Ni insert was significantly reduced. Compared with the P (2 × 2) self-assembly mode, the interaction energy of the PP–DT interface and the PP–DDT interface were increased and the interaction energy of the PP–PFDT interface was decreased. When the self-assembled mode is different, the interaction energy of the PP alkyl mercaptan interface changes greatly, which leads to different adhesion strength between PP and Ni. When the PP–DT interface changes from P (2 × 2) to C (2 × 2), the electrostatic repulsion force changes to electrostatic attraction, as shown in Table 4.

## 5. Conclusions

In this study, alkanethiol was selected as the self-assembled coating on an Ni mold insert. The effect of the alkanethiol coating on the injection-molded microstructures was explored by experiments. Accordingly, the interaction mechanism between alkanethiols and PP was analyzed by MD simulation. The main conclusions were as follows:

(1) Experimental results showed that the self-assembled alkanethiol monolayers would not damage the micro-pillars and the surface roughness of the Ni mold insert. The sunken defects on the top surface of the micro-pillars almost disappeared. The alkanethiol coatings could effectively decrease the adhesion and promote the replication fidelity.
(2) PP molecules were found to be adsorbed on the surface of alkanethiol monolayers in MD simulations, with relative concentration reaching the peak at the interface. After surface treatment, the adhesion work and the interfacial energy that mainly came from the vdW force were significantly reduced. The methyl groups in the PP layer and the hydrogen atom in alkanethiols were the main factors that affect the interfacial interaction.
(3) When alkanethiol molecules were assembled in the P (2 × 2) arrangement, the adhesion at the PP–DT interface was the most contradictory. The PP–PFDT interface had the strongest electrostatic interaction energy due to the strong electronegativity of trifluoromethyl. When in the C (2 × 2) arrangement, PP molecules were not easy to adsorb on the PFDT surface due to the decrease in interaction energy.

## Figures and Tables

**Table 1:** Main process parameters of injection molding experiments.

Mold Temperature (°C)	Melt Temperature (°C)	Injection Velocity (mm^3^/s)	Packing Pressure (MPa)	Packing Time (s)	Cooling Time (s)
100	250	18	140	10	60

**Table 2:** Related modelling parameters of the amorphous polypropylene (PP) system.

Material	Polymerization Degree	Number of Chains	Initial Density (g/cm^3^)	Temperature (K)	Box Size (nm)
PP	30	5	0.9	298	3.24 × 3.24 × 2.24

**Table 3:** Interaction energy and adhesion work in three interface models in P (2 × 2) arrangement.

Interface Model	E_interaction_ (Kcal/mol)	vdW Energy (Kcal/mol)		Electrostatic Energy (Kcal/mol)	Adhesion Work (J/m^2^)
		Repulsive Energy	Dispersive Energy		
PP–Ni	−850.08	881.41	−1731.49	0	0.56
PP–DT	−83.64	79.33	−169.76	6.79	0.06
PP–DDT	−127.48	124.64	−236.08	−16.04	0.08
PP–PFDT	−152.31	95.15	−181.41	−66.05	0.10

**Table 4:** Interaction energy and adhesion work in three interface models in the C (2 × 2) arrangement.

Interface Model	E_interaction_ (Kcal/mol)	vdW Energy (Kcal/mol)		Electrostatic Energy (Kcal/mol)	Adhesion Work (J/m^2^)
		Repulsive Energy	Dispersive Energy		
PP–DT	−133.40	89.98	−218.87	−4.51	0.09
PP–DDT	−186.25	173.14	−342.78	−16.61	0.12
PP–PFDT	−116.40	50.18	−103.70	−62.88	0.08

## Data Availability

Not applicable.
